# Molecular Mechanisms and Pathophysiology of Acute Stroke: Recent Advances and Controversies

**DOI:** 10.3390/cimb46040182

**Published:** 2024-03-27

**Authors:** Teresa Gasull, Adrià Arboix

**Affiliations:** 1 Cellular and Molecular Neurobiology (CMN) Research Group at Germans Trias i Pujol Research Institute, 08916 Badalona, Barcelona, Spain; tgasull@igtp.cat; 2 Cerebrovascular Division, Department of Neurology, Hospital Universitari del Sagrat Cor, Universitat de Barcelona, 08029 Barcelona, Catalonia, Spain

Stroke is a prevalent neurological disorder defined as an abnormality in brain function resulting from the disruption of cerebral circulation [1]. It is a heterogeneous and complex disorder and may be ischemic (80% of cases) or hemorrhagic (about 20%) [2,3,4,5]. Increasing evidence suggests that the brain is exquisitely sensitive to even short-duration ischemia and that multiple mechanisms are involved in the tissue damage that results from cerebral ischemia or hemorrhage [6,7].

This Special Issue aims to present some of the most novel pathophysiological and molecular aspects of acute stroke, emphasizing mainly neurological biomarkers, cerebral neuroprotection, and post-stroke vascular cognitive impairment, which have all seen recent advances. Despite significant progress, several controversies persist in the field of acute stroke mechanisms and pathophysiology.

Thirteen manuscripts were submitted for consideration for this Special Issue, and all were subject to the rigorous Current Issues in Molecular Biology review process. In total, seven papers were accepted for publication and inclusion in this Special Issue. The contributions are listed below:

In this Special Issue, five original articles [contributions 1–3,6,7], one review paper [contribution 4] and one case report with a literature review [contribution 5] were reviewed (Table 1). This issue has brought together a diverse set of research articles that highlight the importance of understanding the molecular signaling pathways involved in stroke.

By examining the latest evidence on this topic, a total of four articles analyzed cerebral infarctions [contributions 3,5–7], ischemic and hemorrhagic stroke was analyzed in one study [contribution 1], and post-stroke cognitive impairment and depressive disturbances were the subject of another two studies [contributions 2,4].

Emphasis is placed on the role of blood biomarkers for the diagnosis and outcome at the onset of an ischemic stroke through the role of vascular endothelial growth factors (VEGFs: VEGF-A, VEGFR-1, and VEGFR-2) in different stroke subtypes [contribution 1] and the clinical relevance of serum thrombomodulin at ischemic stroke onset [contribution 6]. The role of the indices of the hypothalamic–pituitary–adrenal axis and inflammatory system in post-stroke vascular-type cognitive impairment and depressive disturbances [contribution 2] and neuroprotection against cerebral ischemia-induced damage [contribution 3] and the neuroprotective activity of melanocortin-like ACTH(4-7)PGP and ACTH(6-9)PGP peptides in rat brains after transient middle cerebral artery occlusion [contribution 7] were also analyzed. Finally, a case report on ischemic stroke due to a rupture of a hydatid cyst, which is a very infrequent cause of ischemic stroke of an unusual etiology subtype, was also reported with an updated review [contribution 5].

The goal of this monographic Issue is to provide a critical overview of the underlying factors involved in stroke-related brain injury, especially the role of blood biomarkers and their recent advances and controversies. Babkinia et al. [contribution 1] characterize the changes in serum levels of vascular endothelial growth factors, which are important regulators of angiogenesis, neuroprotection, and neurogenesis. The authors analyzed the changes in serum levels of VEGF-A, VEGFR-1, and VEGFR-2 in patients at various phases of ischemic and hemorrhagic strokes. The authors identified different levels of VEGF-A and its receptors at various phases of acute stroke. They remarked that, in ischemic stroke, increased VEGFR-2 levels were found in the hyperacute and acute phases. Levels of VEGF-A were elevated in the early subacute phase. In the early subacute phase, reduced VEGFR-1 levels were revealed. In the acute and early subacute phases of hemorrhagic stroke, there was an increase in the levels of VEGF-A and VEGFR-2.

Cognitive decline after stroke is another relevant feature in stroke patients. In an original article, Zhanina et al. [contribution 2] analyzed the relationship between neurohumoral indices and the development of neuropsychiatric complications, including, in particular, post-stroke cognitive and depressive disturbances within a year following ischemic stroke. These neurohumoral problems complicates the rehabilitation, quality of life, and social adaptation of patients. The results suggest the prolonged hyperactivation of the hypothalamic–pituitary–adrenal (HPA) axis and sympathoadrenal medullary system after ischemic stroke. Post-stroke cognitive impairment was associated with the hyperactivation of the HPA axis during the acute ischemic stroke period, while post-stroke depressive disorder was associated with the chronic inflammatory process and hyperactivation of the sympathoadrenal medullary system during the follow-up period.

Lee H.-G. et al. [contribution 3], using an in vivo experimental stroke model (permanent middle cerebral artery occlusion in a male mice model), analyzed the neuroprotective effects of Geopung-Chunghyuldan (GCD) based on its salvianolic acid B content. The authors found that GCD with high salvianolic acid B, but not GCD with low salvionic acid, showed marked neuroprotective effects compared to the control groups. Because an in vivo model was used in this study, the additional verification of the evidence presented in this preliminary study is necessary through future clinical trials.

In a review paper, Colita et al. [contribution 4] summarized the recent literature reporting the role of the therapeutic use and chronic abuse of central nervous system stimulants and anabolic drugs. Most of these substances can have deleterious brain-related side effects, and doping may cause serious health-threatening conditions, including cardiovascular diseases, cerebral thrombosis, cognitive decline or subdural hematomas.

In their case report, Lungu et al. [contribution 5] reported a 25-year-old patient with an acute stroke in the left posterior cerebral artery associated with acute limb ischemia due to the rupture of a hydatid cyst. Human cystic echinococcosis is a neglected zoonotic disease present in endemic areas (like the Mediterranean zone) and has been increasing due to population migration with various and heterogeneous clinical manifestations. An embolism in patients with ruptured hydatid cysts can be the cause of an ischemic stroke of an unusual etiology subtype. Surgery with medical therapy comprising benzimidazole drugs (albendazole +/− praziquantel) is considered the standard of care, aiming for scolicidal and anti-cystic activity.

Moreover, the original article by Zaharia et al. [contribution 6] shows the potential utility of thrombomodulin (TM) as a biomarker for stroke. TM is a type-1 transmembrane glycoprotein with primary expression in endothelial cells that plays an important role in a multitude of processes, with biological functions attributed to various subdomains of soluble TM. The authors found that acute ischemic stroke patients presented a significant increase in TM values in the first 24 h after onset. Moreover, serum levels correlated with the severity of acute ischemic stroke development and the risk of death, and were influenced by associated cardiovascular risk factors.

Lastly, Stavchansky V.V. et al. [contribution 7], using an in vivo experimental stroke model, analyzed the neuroprotective activity of melanocortin-like ACTH(4-7)PGP and ACTH(6-9)PGP peptides in rat brains after transient middle cerebral artery occlusion. The authors show a significant increase in the proliferative activity of neuroglial cells and the vascularization of brain tissues in the perifocal ischemic zones and the penumbra after the administration of ACTH(4-7)PGP and ACTH(6-9)PGP. These findings confirm the possible protective effect of these peptides.

Overall, the research presented in this Special Issue entitled “Molecular Mechanisms and Pathophysiology of Acute Stroke” underscores the complex nature of strokes and the importance of a multidisciplinary research approach to gain an understanding of stroke pathophysiology [7,8,9,10]. In conclusion, this Special Issue highlights a number of research directions and topics addressed to study the molecular and cellular mechanisms of acute stroke. Improving our understanding of the underlying molecular mechanisms of cerebrovascular diseases is imperative for the further incorporation of innovative drugs and cell and gene therapy approaches into carefully controlled clinical trials and could promote significant advances in a field with many unmet needs. We extend our sincere thanks to all the authors, reviewers, and editors who contributed to this Special Issue.

## Figures and Tables

**Table 1:** Analysis of the published contributions included in the present Special Issue “Molecular Mechanisms and Pathophysiology of Acute Stroke”.

N#*	Authors	I/H	Stroke Relevance	Topic	Characteristic of the Article	Conclusions
1	Babkina et al.	I/H	Hemorrhagic and ischemic stroke pathophysiology	The role of vascular endothelial growth factors (VEGFs: VEGF-A, VEGFR-1, and VEGFR-2) in stroke subtypes was studied.	Retrospective cohort clinical study	In ischemic stroke, an increased VEGFR-2 level was found in the hyper-acute and acute phases, while elevated VEGF-A and reduced VEGFR-1 levels were revealed in the early subacute phase. In hemorrhagic stroke, no significant changes in the levels of VEGF-A and its receptors were identified in the hyper-acute phase. In the acute and early subacute phases, there was an increase in levels of VEGF-A and VEGFR-2, respectively.
2	Zhanina et al.	I	Post-stroke cognitive impairment and depressive disturbances	Role of the indices of the HPA (hypothalamic–pituitary–adrenal) axis, inflammatory system, and SAMS in blood serum (cortisol, interleukin-6 (IL-6), plasma (adrenocorticotropic hormone), and saliva (cortisol, α-amylase) and its relation to post-stroke cognitive impairment and post-stroke depressive disorder.	Single-center prospective clinical study	Post-stroke cognitive impairment was associated with the hyperactivation of the hypothalamic–pituitary–adrenal axis during the acute ischemic stroke period, while post-stroke depressive disorder was associated with the chronic inflammatory process and hyperactivation of the sympathoadrenal medullary system during the follow-up period.
3	Lee H.-G. et al.	I	Neuroprotection in ischemic stroke	Evaluation of the neuroprotective effects of Geopung-Chunghyuldan (GCD) based on salvianolic acid B content in experimental stroke	Experimental in vivo stroke study	The salvianolic acid B content of *Salviae miltiorrhizae* Radix affects the neuroprotection and effect of Geopung-Chunghyuldan.
4	Coliță D. et al.	-	Cognitive decline	Impact of doping on psychopathological disorders, cognition, and depression	Updated review	Raising awareness of the health risks of doping in sports for all could promote an increased awareness for healthy lifestyles across all generations.
5	Lungu M. et al.	I	Ischemic stroke of unusual etiology	Ischemic stroke due to rupture of a hydatid cyst	Case report and literature review	The rupture of a hydatid cyst can cause embolism with anhistous membranes with ischemia in the right brachial artery and left posterior cerebral artery occlusion, which is associated with anaphylactic phenomena. It is an unusual etiology of acute ischemic stroke.
6	Zaharia, A.-L. et al.	I	Biomarker for the acute onset of ischemic stroke	Clinical relevance of serum levels of thrombomodulin	Observational, prospective, clinical, and analytical monocentric study.	Serum thrombomodulin levels may represent a potentially valuable biomarker for the diagnosis of the onset of an ischemic stroke, even with scarce clinical neurological manifestations. Moreover, serum levels correlated with the severity of ischemic stroke development and the risk of death are influenced by associated cardiovascular risk factors.
7	Stavchansky V.V. et al.	I	Neuroprotection in ischemic stroke	Neuroprotective activity of melanocortin-like ACTH(4-7)PGP and ACTH(6-9)PGP peptides in rat brains after transient middle cerebral artery occlusion.	Experimental in vivo stroke study	A significant increase in the volume density of vessels and their sizes in the penumbra was observed after the administration of ACTH(4-7)PGP and ACTH(6-9)PGP. These findings confirm the neuroprotective effect of peptides due to the activation of neuroglia proliferation and the enhancement of collateral blood flow.

I: ischemic stroke/H: hemorrhagic stroke; N#*: number of contributions.
